# Dissecting the Role of Curcumin in Tumour Growth and Angiogenesis in Mouse Model of Human Breast Cancer

**DOI:** 10.1155/2015/878134

**Published:** 2015-03-23

**Authors:** Sabrina Bimonte, Antonio Barbieri, Giuseppe Palma, Domenica Rea, Antonio Luciano, Massimiliano D'Aiuto, Claudio Arra, Francesco Izzo

**Affiliations:** ^1^Hepatobiliary Unit, Istituto Nazionale per la Studio e la Cura dei Tumori “Fondazione G. Pascale” (IRCCS), Via Mariano Semmola, 80131 Naples, Italy; ^2^S.S.D. Sperimentazione Animale, Istituto Nazionale per lo Studio e la Cura dei Tumori “Fondazione G. Pascale” (IRCCS), Via Mariano Semmola, 80131 Naples, Italy; ^3^Istituto di Endocrinologia e Oncologia Sperimentale del Consiglio Nazionale delle Ricerche c/o, Dipartimento di Biologia e Patologia Cellulare e Molecolare “L. Califano”, Università degli Studi di Napoli “Federico II”, Via Pansini 5, 80131 Naples, Italy; ^4^Division of Breast Surgery, Department of Breast Disease, Istituto dei Tumori di Napoli “Fondazione G. Pascale” (IRCCS), 80131 Naples, Italy

## Abstract

Breast cancer is considered the most common cancer for women worldwide and it is now the second leading cause of cancer-related deaths among females in the world. Since breast cancer is highly resistant to chemotherapy, alternative anticancer strategies have been developed. In particular, many studies have demonstrated that curcumin, a derivative of turmeric, can be used as natural agent in treatment of some types of cancer by playing antiproliferative and antioxidant effects. In our study, we assessed the antitumor activities of curcumin in ER-negative human breast cancer cell line resistant to chemotherapy, MDA.MB231 by *in vitro* and *in vivo* experiments. *In vitro* data allowed us to demonstrate that curcumin played a role in regulation of proliferation and apoptosis in MDA.MB231 cells. *In vivo*, by generation of mouse model of breast cancer, we showed that treatment of curcumin inhibited tumor growth and angiogenesis. Specifically, we showed that curcumin is able to deregulate the expression of cyclin D1, PECAM-1, and p65, which are regulated by NF-*κ*B. Our data demonstrated that curcumin could be used as an adjuvant agent to chemotherapy in treatment of triple negative breast cancer.

## 1. Introduction

Breast cancer is considered the most common cancer for women worldwide and it is now the second leading cause of cancer-related deaths among females in the world [1]. Since breast cancer is highly resistant to chemotherapy [2, 3] and in particular very limited treatment options exist for the ER-negative patients [4], alternative anticancer strategies are needed. Curcumin, which is extracted from the plant* Curcuma longa*, is such natural agent with anti-antitumor and anti-inflammatory effects. Several preclinical studies focused on the anticancer efficacy of curcumin have been tested in some cancer models including breast cancer [5]. Results from these studies reported that curcumin alone, in combination with targeted compounds or chemotherapeutic agents, is able to inhibit human cancer cell proliferation and tumorigenesis at different molecular levels [6–15]. Many studies have reported that curcumin inhibits human breast cancer cell growth by modulating the NF-*κ*B signaling pathway [11, 13, 16–20]. In addition, it has been showed that curcumin decreased human epidermal growth factor 2 (HER2) oncoprotein expression, the phosphorylation of Akt, MAPK, and the expression of NF-*κ*B in both BT-474 and SK-BR-3-hr cells [21]. Interestingly, it has been found that curcumin suppressed breast tumor angiogenesis by abrogating osteopontin or medroxyprogesterone acetate induced VEGF expression [22, 23]. Moreover, it has been reported that the combination of epigallocatechin gallate (EGCG) and curcumin is efficacious in both* in vitro* and* in vivo* models of ER*α*-breast cancer by the regulation of VEGFR-1 expression [24]. The effects of curcumin have been also tested in MDA.MB231 cells by different groups [25–32]. In our study, we dissected the role of curcumin on tumour growth and angiogenesis in mouse model of human breast cancer. We first demonstrated that curcumin,* in vitro*, has a role in the regulation of proliferation and apoptosis of MDA.MB231 cells.* In vivo*, we showed that curcumin inhibited tumor growth and angiogenesis in a heterotopic mouse model of breast cancer by influencing the expression of NF-*κ*B-regulated gene products (cyclin D1, PECAM-1, and p65). Taken together, our data indicate that curcumin could be used as an adjuvant agent to chemotherapy in treatment of triple negative breast cancer.

## 2. Material and Methods 

### 2.1. Reagents

Curcumin, used for* in vitro* experiments, was obtained from Sigma Aldrich (Piscataway, NJ) and was dissolved in dimethyl sulphoxide (DMSO) to a concentration of 500 *μ*M as a stock solution. It was diluted in DMEM and 10% FBS and added to MDA.MB231 cells in two different doses (10 and 50 *μ*M). The following polyclonal antibodies against cyclin D1, and polyclonal antibodies against PECAM-1, were obtained from Santa Cruz Biotechnology (Santa Cruz, CA). Anti-p65 antibody was kindly provided by Imgenex (San Diego, CA). The liquid DAB+ Substrate Chromogen System-HRP used for immunocytochemistry was obtained from DakoCytomation (Carpinteria, CA). Penicillin, streptomycin, RPMI 1640, and fetal bovine serum (FBS) were obtained from Invitrogen (Grand Island, NY). Tris, glycine, NaCl, SDS, and bovine serum albumin (BSA) were obtained from Sigma Chemical (St. Louis, MO). Complete feed for mice with curcumin 0, 6% (AIN-93G) was purchased by Mucedola (Settimo Milanese, Italy).

### 2.2. Cell Lines and Mice

ER-negative breast cancer cell line MDA.MB231 was obtained from American Type Culture Collection (Manassas, VA). Cells were cultured in Dulbecco's Modified Eagle Medium (DMEM) supplemented with fetal bovine serum (FBS) 10%, antibiotics (penicillin 100 units/mL, streptomycin 100 *μ*g/mL), and l-glutamine (2 mM) at 37°C in atmosphere of 5% of CO_2_. Female Foxn1^nu/nu^ mice (six to eight weeks old, Harlan, San Pietro al Natisone) were maintained under specific pathogen free (SPF) conditions.

### 2.3. Proliferation Assay, Wound-Healing Assay, and* In Vitro* Apoptosis

The effect of curcumin on cell proliferation was determined by using TACS 3-(4,5-dimethylthiazol-2-yl)-2,5-diphenyltetrazolium bromide (MTT) cell proliferation assay (Trevigen, Gaithersburg) as previously described [15]. Wound-healing assay and* in vitro* apoptosis on MDA.MB231 cells were performed as previously described [15, 33].

### 2.4. NF-*κ*B Activation in Cell and Tumor Samples

To assess NF-*κ*B activation, we performed electrophoresis mobility shift assays (EMSA) as previously described [15, 33].

### 2.5. Western Blot Analysis

Breast tumor tissues (75–100 mg/mouse) from control and mice treated with curcumin were minced and incubated on ice for 1 h in 0.5 mL of ice-cold Lysis Buffer (10 mM Tris, pH 8.0, 130 mM Nail, 1% Triton X-100, 10 mM NaF, 10 mM sodium phosphate, 10 mM sodium pyrophosphate, 2 *μ*g/mL aprotinin, 2 *μ*g/mL leupeptin, and 2 *μ*g/mL pepstatin). The minced tissues were homogenized using a Dounce homogenizer and centrifuged at 16,000 ×g at 4°C for 10 min. Western blotting analysis was performed according to standard protocols. *α*-actin and *β*-tubulin were used as loading control.

### 2.6. Establishment of Subcutaneous Xenograft Breast Tumor Model in Nude Mice

The nude mice were subcutaneously inoculated with cell suspension containing 2,5 × 10^6^ MBA.MB231 cells into the right side flank area of mice. A total of 16 female Foxn1^nu/nu^ mice were used in this experiment and maintained in a barrier facility on HEPA-filtered racks. The subcutaneous tumors were monitored and when the volume of tumors reached ~30–60 mm^3^, mice were randomized into the following treatment groups (*n* = 2): (a) 8 untreated mice placed in normal diet and (b) 8 mice treated with curcumin placed in diet containing curcumin at 0,6%. Tumor volumes were monitored once a week by using a digital caliper. Therapy was continued for 6 weeks and animals were sacrificed at reaching of cut-off. Measured values were used to calculate the tumor volume according to the formula [length (mm) × width (mm)^2^]/2. In order to test microvessel formation on tumor tissues in mice, fluorescein-isothiocyanate- (FITC-) dextran (100 *μ*L) was injected into the tail veins of mice to visualize microvessels within 150 *μ*m (using single-photon microscopy) or ~600 *μ*m (using multiphoton laser-scanning microscopy [MPLSM]) of a tumour/window interface. After sacrifice of mice, a portion of the tumor tissue was fixed in 10% formalin for histological analysis while the remaining tissue was stored at −80°C for molecular studies. All the experiments performed on mice were in compliance with the guidelines for the Care and Use of Laboratory Animals of the National Cancer Institute, IRCCS, Fondazione Pascale.

### 2.7. Immunohistochemistry

Tumour samples from controls and treated-mice were embedded in paraffin and fixed with paraformaldehyde. After being washed in PBS, the slides were blocked with protein block solution (DakoCytomation) for 20 min and then incubated overnight with polyclonal anti-goat PECAM-1 (1 : 100). After the incubation, the slides were washed and then incubated with biotinylated link universal antiserum followed by horseradish peroxidase-streptavidin conjugate (LSAB + kit). The slides were rinsed, and colour was developed using 3,3′-diaminobenzidine hydrochloride as a chromogen. Finally, sections were rinsed in distilled water, counterstained with haematoxylin, and mounted with DPX mounting medium for evaluation. Pictures were captured with a Photometric Cool SNAP CF colour camera (Nikon, Lewisville, TX) and MetaMorph version 4.6.5 software (Universal Imaging, Downingtown, PA).

### 2.8. Statistical Analysis

Statistical analysis was performed using SPSS13.0 software. Experiments were repeated at least three times with consistent results. Normally distributed data were represented as mean ± SEM. Paired* t-*test was used to examine the significance of differences among groups (GraphPad Prism 5.0). A probability value with ^*^ *P* < 0.05 and ^**^ *P* < 0.01 was considered to be statistically significant.

## 3. Results

### 3.1. Curcumin Has a Role in the Regulation of Proliferation and Apoptosis of MDA.MB231 Breast Cancer Cells

**Figure 1:**
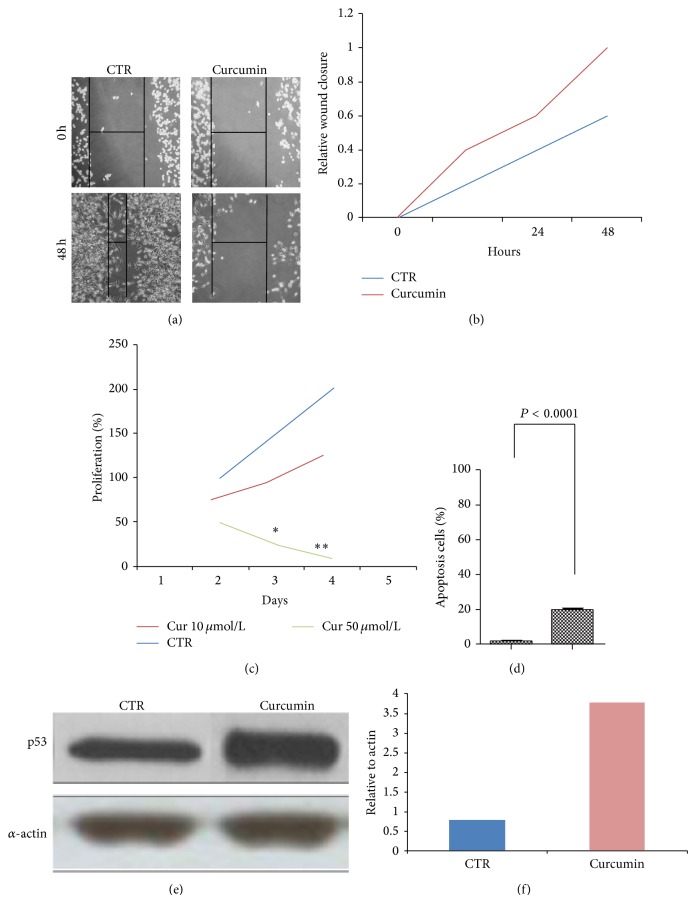
Curcumin inhibits proliferation and enhances apoptosis in MDA.MB231 cancer cells. (a) For wound-healing assay, MDA.MB231 cells were wounded by scratching and monitored over 48 h to determine the rate of wound closure (40x magnification), scale bar, 200 *μ*m. (b) Cell migration was assessed by measuring relative wound closure. Data represent mean ± SEM (^**^ *P* < 0.01; ^*^ *P* < 0.05). At 48 h after wound induction, there were clearly less cells in the denuded area of curcumin treated cells than untreated cells. (c) MTT assay results show a suppression of proliferation in breast cancer cells treated with curcumin respect to control cells S.E. Data represent mean ± SEM (^**^ *P* < 0.01; ^*^ *P* < 0.05). (d)* In vitro* apoptosis assay by flow cytometry indicated that curcumin (10 *μ*M) enhances apoptosis in MDA.MB231 cells (*P* value < 0.0001). ((e)-(f)) Western blot showing that curcumin enhances the expression of p53 in MDA.MB231 cells treated with curcumin (lane 2) with respect to controls (lane 1). *α*-actin was used as loading control.

In order to assess the effects of curcumin on proliferation and apoptosis of MBA.MB231 cells, we performed* in vitro* assays. Results from wound-healing and MTT tests showed that curcumin inhibits the migration of breast cancer cells at 48 h (Figures 1(a), 1(b), and 1(c)). By performing flow cytometry analysis, we demonstrated that curcumin regulates the apoptosis process of MBA.MB231 cells (Figure 1(d)). This result was also confirmed by Western blotting analysis of p53 expression (Figures 1(e) and 1(f)). Taken together, our results suggest that curcumin inhibits proliferation and enhanced apoptosis of MDA.MB231 breast cancer cells.

### 3.2. Curcumin Inhibits the Tumour Growth and Microvessel Formation in Heterotopic Mouse Model of Breast Cancer

**Figure 2:**
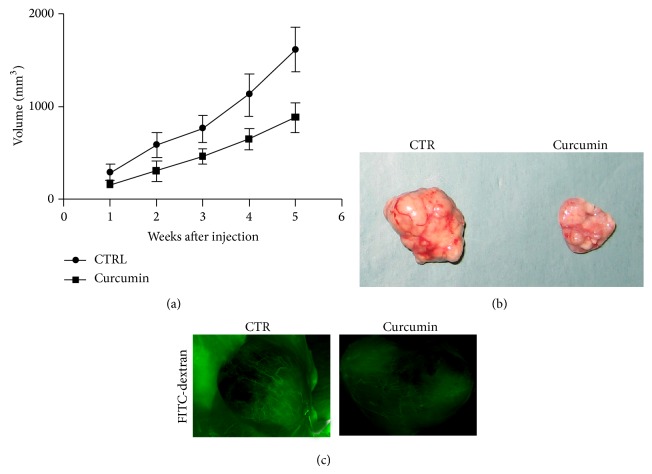
Curcumin inhibits the tumor growth in xenograft mouse model of breast cancer. (a) Curcumin inhibits tumor growth in breast tumor xenograft model. Breast tumor growth in 8 mice treated with vehicle (•) and 8 mice treated with curcumin (■). Tumor volumes decreased significantly after 3 weeks of curcumin treatment until 6 weeks (*P* = 0.0195) as compared with control (vehicle-treated). (b)* Ex vivo* tumour from control (left) and treated mice (right). (c) Measurements of fluorescence per second depicting microvessel tumor (FITC-DEXTRANE) using MacroFluo images showed that curcumin inhibits the angiogenesis in tumour of mice treated with curcumin (right) with respect to controls (left).

We generated a mouse model of breast cancer, to study the role of curcumin on tumor growth and angiogenesis. When tumors reached ~30–60 mm^3^, 16 mice were randomized into two groups: 8 controls (normal diet) and 8 curcumin-treated. The treatment with curcumin started after tumor cell's implantation and continued up to 6 weeks. Results obtained by detection of mice body indicated that curcumin has no toxicity effect on mice. Tumor volumes decreased significantly after 3 weeks of curcumin treatment until 6 weeks as compared to controls (Figures 2(a) and 2(b)). In order to assess if curcumin inhibits microvessel formation in breast tumors, fluorescein-isothiocyanate- (FITC-) dextran was injected into the tail veins of mice. Our data demonstrate that curcumin reduces microvessel formation in tumours of treated mice with respect to controls (Figure 2(c)).

### 3.3. Effects of Curcumin on NF-*κ*B Activation in Heterotopic Breast Tumors

**Figure 3:**
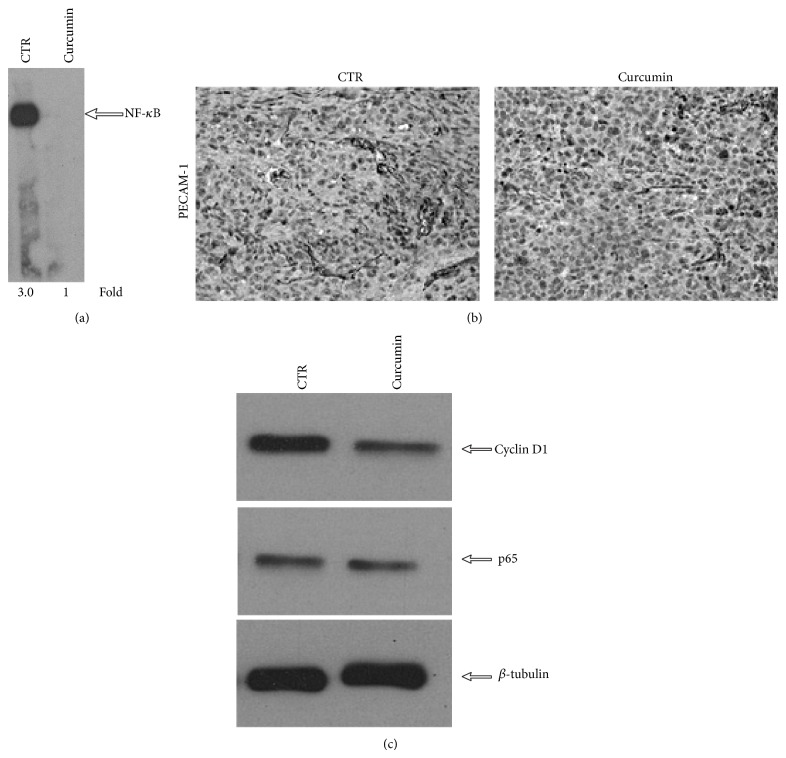
Curcumin inhibits NF-*κ*B activation and downregulates NF-*κ*B-regulated gene products in breast tumors. (a) EMSA assay performed on tumor tissue samples showed the inhibition of NF-*κ*B by curcumin. (b) Immunohistochemistry for PECAM-1 showed the inhibition of PECAM-1 expression in curcumin-treated group, compared to controls. (c) Western blot showing that curcumin inhibits the expression of NF-*κ*B–dependent gene products, cyclin D1, and p-65 in breast tumor tissues. Samples from three animals in each group were analyzed and representative data are shown. *β*-tubulin was used as loading control.

It has been reported that curcumin potentiates the antitumor activity of paclitaxel in breast cancer by influencing the expression of NF-*κ*B-regulated gene products [34]; for this reason we performed DNA binding assay on tumors of control and curcumin-treated mice. Our results showed that curcumin inhibits the activation of NF-*κ*B (Figure 3(a), lane 2). We also tested the effects of curcumin on the expression of NF-*κ*B-regulated gene products such as cyclin D1, p65, and PECAM-1, by immunohistochemistry and Western blotting analysis. Our results showed that in tumours of curcumin-treated group, there were significant reductions in the expression of PECAM-1, cyclin D1, and p65 compared to the control group (Figures 3(b) and 3(c)). These data showed that curcumin is able to influence the expression of NF-*κ*B-regulated gene products in mouse model of breast cancer.

## 4. Discussion

In this paper we dissected the role of curcumin in tumor growth and angiogenesis in mouse model of human breast cancer.* In vitro* data allowed us to demonstrate that curcumin regulates the proliferation and the apoptosis of MDA.MB231 cells. To test the effects of curcumin in heterotopic mouse models of breast cancer, we used complete feed for mice with curcumin 0,6%. Based on mice diet tables reported in literature [35], we have estimated, approximatively, that mice assumed 25 mg/die of curcumin. We decided to use this system also to overcome the problem of low* in vivo* bioavailability of curcumin [36].* In vivo* data showed that tumors of mice treated with curcumin were smaller than those observed in controls, indicating that curcumin has an antitumor effect on breast cancer cells. We showed that curcumin inhibits tumor growth and angiogenesis through the modulation of NF-*κ*B pathway. Since curcumin is very well tolerated in human subjects and is assumed by food, our data demonstrated that curcumin could be considered an alternative nontoxic agent in the treatment of triple negative breast cancer.
